# Retraction: The influence of machine learning-based knowledge management model on enterprise organizational capability innovation and industrial development

**DOI:** 10.1371/journal.pone.0321413

**Published:** 2025-04-09

**Authors:** The PLOS One Editors

The *PLOS One* Editors retract this article [1] because it was identified as one of a series of submissions for which we have concerns about peer review integrity and potential manipulation of the publication process. These concerns call into question the validity and provenance of the reported results. We regret that the issues were not identified prior to the article’s publication.

All authors either did not respond directly or could not be reached.

## References

[1] Zhou Z, Liu Y, Yu H, Ren L. The influence of machine learning-based knowledge management model on enterprise organizational capability innovation and industrial development. PLoS One. 2020;15(12): e0242253. doi: 10.1371/journal.pone.0242253 33259475 PMC7707476

